# Hydrogel particles improve detection of SARS-CoV-2 RNA from multiple sample types

**DOI:** 10.1038/s41598-020-78771-8

**Published:** 2020-12-30

**Authors:** R. A. Barclay, I. Akhrymuk, A. Patnaik, V. Callahan, C. Lehman, P. Andersen, R. Barbero, S. Barksdale, R. Dunlap, D. Goldfarb, T. Jones-Roe, R. Kelly, B. Kim, S. Miao, A. Munns, D. Munns, S. Patel, E. Porter, R. Ramsey, S. Sahoo, O. Swahn, J. Warsh, K. Kehn-Hall, B. Lepene

**Affiliations:** 1 Ceres Nanosciences, Inc., Manassas, VA 20110 USA; 2 National Center for Biodefense and Infectious Diseases, School of Systems Biology, George Mason University, Manassas, VA 20110 USA

**Keywords:** Biological techniques, Biotechnology, Microbiology

## Abstract

Here we present a rapid and versatile method for capturing and concentrating SARS-CoV-2 from contrived transport medium and saliva samples using affinity-capture magnetic hydrogel particles. We demonstrate that the method concentrates virus from 1 mL samples prior to RNA extraction, substantially improving detection of virus using real-time RT-PCR across a range of viral titers (100–1,000,000 viral copies/mL) and enabling detection of virus using the 2019 nCoV CDC EUA Kit down to 100 viral copies/mL. This method is compatible with commercially available nucleic acid extraction kits (i.e., from Qiagen) and a simple heat and detergent method that extracts viral RNA directly off the particle, allowing a sample processing time of 10 min. We furthermore tested our method in transport medium diagnostic remnant samples that previously had been tested for SARS-CoV-2, showing that our method not only correctly identified all positive samples but also substantially improved detection of the virus in low viral load samples. The average improvement in cycle threshold value across all viral titers tested was 3.1. Finally, we illustrate that our method could potentially be used to enable pooled testing, as we observed considerable improvement in the detection of SARS-CoV-2 RNA from sample volumes of up to 10 mL.

## Introduction

Severe acute respiratory syndrome coronavirus 2 (SARS-CoV-2) is a member of the Coronaviridae family and is responsible for the pandemic outbreak of coronavirus disease 2019 (COVID-19) that emerged in December 2019 in Wuhan, Hubei Province, China^1^. The disease is characterized by fever, dry cough, fatigue, anorexia, shortness of breath, and myalgia^2,3^. COVID-19 rapidly spread and by March 11, 2020, the World Health Organization (WHO) declared COVID-19 a global pandemic^4^. As of June 7, 2020, there have been more than 7 million COVID-19 cases and 411,177 deaths worldwide^5^. Such a fast-acting and massive outbreak throughout the world has led to severe impacts on the health care systems and economies of countries around the globe.

The rapid spread of SARS-CoV-2 led to a tremendous increase in demand for COVID-19 diagnostic testing worldwide. At present, the United States Centers for Disease Control and Prevention (CDC) recommends diagnosis of acute SARS-CoV-2 infection via measurement of viral nucleic acid and antigen tests (https://www.cdc.gov/coronavirus/2019-nCoV/hcp/clinical-criteria.html). Real-time reverse transcriptase-polymerase chain reaction (real-time RT-PCR) is a reliable and relatively fast method for the identification of pathogenic nucleic acids in patient samples^6^. However, it has several disadvantages. High quality RNA is critical to real-time RT-PCR assays. Thus, the initial step often involves purification of SARS-CoV-2 RNA from patient samples, (i.e. nasopharyngeal swabs, saliva, or bronchoalveolar lavages) using a phenol/chloroform-based method or commercial RNA purification kits, both of which have multiple, time consuming steps and can contain dangerous reagents. Commercial RNA purification kits often rely on column-based isolation, which can take over 30 min and requires a centrifugation or vacuum filtration step, or magnetic bead isolation, have multiple steps, and are limited by the initial amount of starting material that can be added to the beads in a plate-based format^7^. Moreover, in recent months, the manufacturers of these kits have struggled to keep up with demand, resulting in shortages in the United States^8^. Another disadvantage is the requirement for relatively high concentrations of genetic material in a sample. Although real-time RT-PCR is considered to be a sensitive method for the detection of nucleic acid, the limit of detection for SARS-CoV-2 RNA is reported to be between 200 and 77,440 copies/mL, depending on the RNA extraction method and real-time RT-PCR assay being used^6,9,10^.

We sought to address these issues with a method, based on our affinity-capture hydrogel particles (Nanotrap particles), that captures and concentrates SARS-CoV-2 from samples prior to RNA purification to improve detection of the virus when used with CDC-recommended SARS-CoV-2 assays. Here, we demonstrate that a 5-min Nanotrap particle capture step substantially increases the sensitivity of SARS-CoV-2 real-time RT-PCR assays when used in conjunction with either commercial RNA extraction kits or a simple heat and detergent extraction method in both saliva and transport medium samples. Furthermore, with this method we identified viral RNA in several diagnostic remnant samples that previously had tested negative for SARS-CoV-2. Finally, we tested and confirmed the ability of a Nanotrap particle method to improve detection of SARS-CoV-2 in pooled patient sample mimics, a promising approach that shows potential for addressing the massive testing scale-ups that are necessary. Taken together, the methods here are quick and easy to implement, requiring only a magnetic tube rack to separate the particles and captured viruses, and substantially improve the detection capability of current SARS-CoV-2 test strategies.

## Results

### Nanotrap particles capture and concentrate live SARS-CoV-2 from transport medium

In previous studies, we have shown that our hydrogel particles (Nanotrap particles) functionalized with high-affinity chemical baits capture and concentrate a variety of respiratory viruses from biological samples, including Influenza A and B, RSV, and Coronavirus 229-E^11–14^. In light of this, we asked whether the same particles could capture and concentrate SARS-CoV-2 from transport medium in order to improve diagnostic testing for COVID-19.

**Figure 1:**
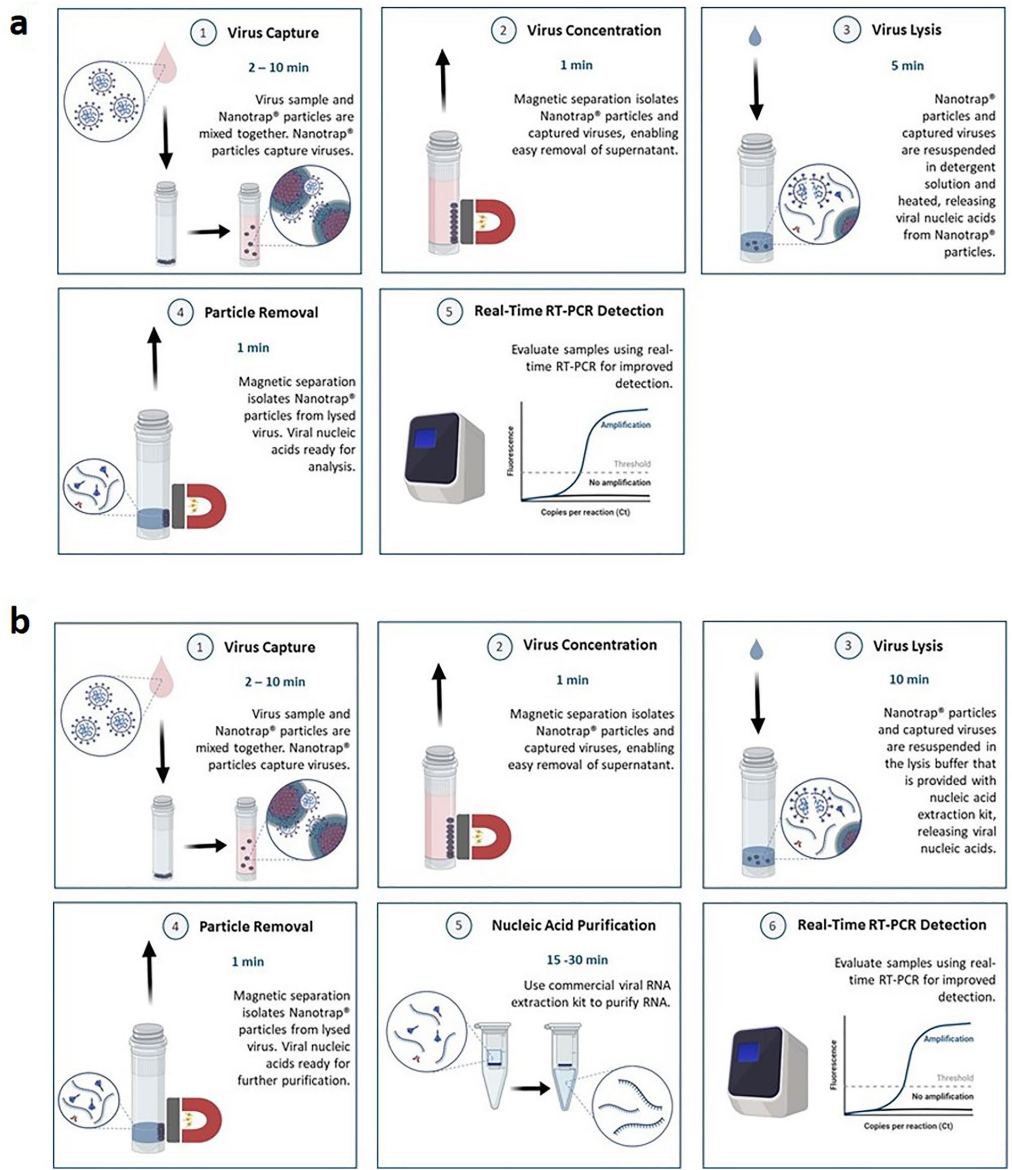
Nanotrap particles are compatible with multiple extraction methods. Sample is mixed with Nanotrap particles and incubated for 2–10 min. Nanotrap particles are pelleted with a magnet, the supernatant is removed, and the particle pellet is resuspended in a viral lysis solution. The Nanotrap particles are pelleted with a magnet, and (**a**) the supernatant can be used directly in real-time RT-PCR (total workflow time is 10–20 min) or (**b**) the supernatant can be processed with a commercial RNA extraction kit prior to viral detection (total workflow time is 30–50 min.)

In all experiments, a simple four-step workflow was used to capture and concentrate the virus using our Nanotrap particles. Briefly, viral transport medium (VTM) spiked with heat-inactivated SARS-CoV-2 was mixed with Nanotrap particles and incubated at room temperature. The Nanotrap particles and the captured viruses were separated from the solution using a magnet and the supernatant was removed. The particle pellet was re-suspended in lysis buffer, which lysed the virus and released its nucleic acid. The Nanotrap particles were pelleted a second time with a magnet, and the nucleic acid-containing supernatant was ready for analysis by real-time RT-PCR (if using a direct nucleic acid extraction method, Fig. 1a) or for further RNA purification (if using a commercial nucleic acid kit extraction method, Fig. 1b).

**Figure 2:**
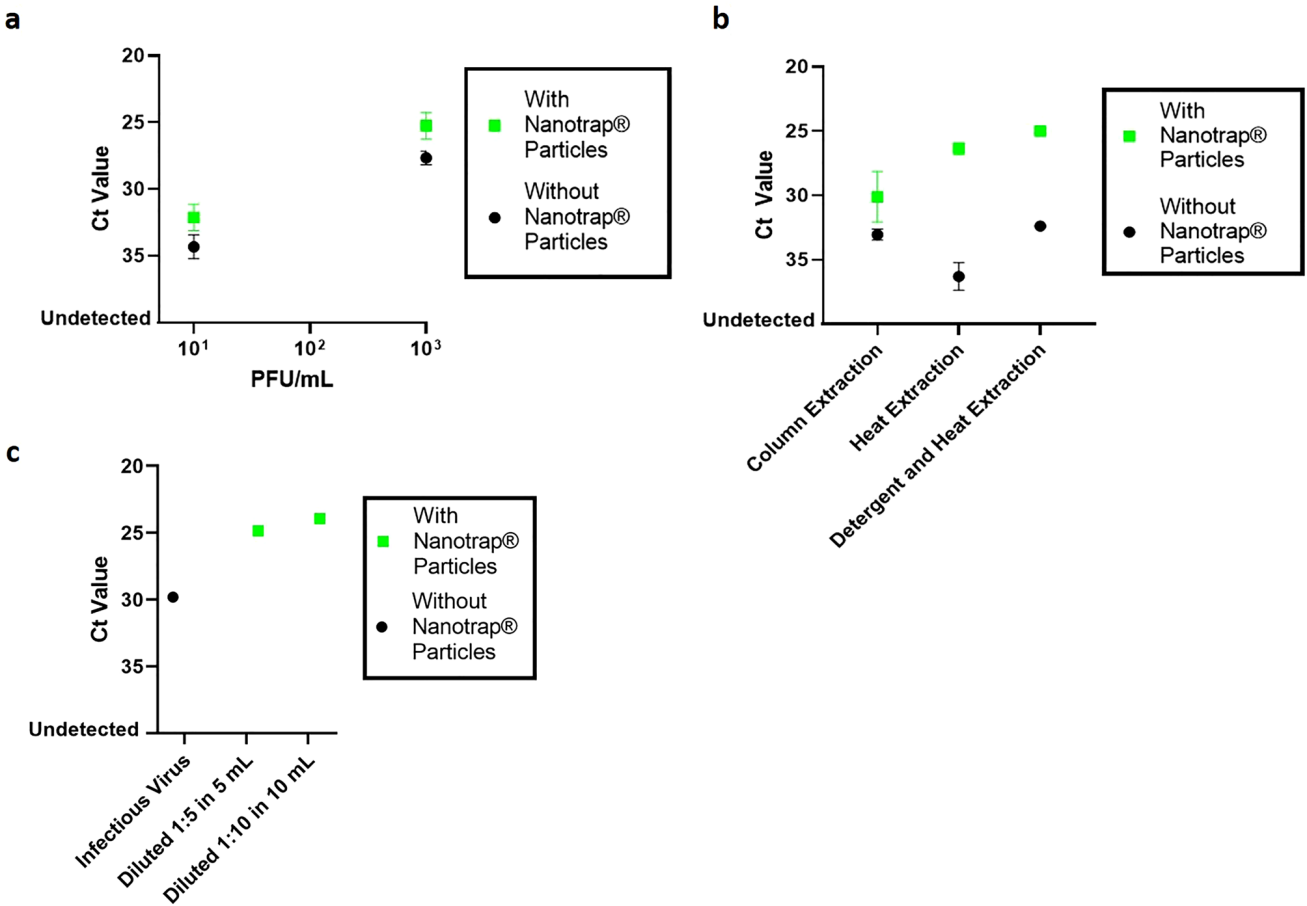
Nanotrap particles capture infectious SARS-CoV-2 from transport medium and improve detection by real-time RT-PCR. (**a**) SARS-CoV-2 was spiked into viral transport medium at various titers. One milliliter samples were added to Nanotrap particles and incubated for 30 min. Viral extraction was performed using TRIzol LS and the RNeasy Kit, and viral detection was performed by real-time RT-PCR. Samples without Nanotrap particles were processed as controls. (**b**) SARS-CoV-2 was spiked into viral transport medium at 1000 PFU/mL. One milliliter samples were added to Nanotrap particles and incubated for 30 min. Viral extraction was performed using the RNeasy Kit, heat extraction, or detergent and heat extraction methods. Viral RNA was detected by real-time RT-PCR. (**c**) SARS-CoV-2 was spiked into 1 mL of transport medium at 100 PFU/mL. Virus spiked-medium was mixed with 4 or 9 mL of uninfected transport medium in the presence or absence of Nanotrap particles. The samples without Nanotrap particles were processed using the RNeasy kit. Samples with Nanotrap particles were incubated for 30 min prior to heat and detergent extraction. Viral RNA was detected using real-time RT-PCR. Samples without Nanotrap particles were used as controls. Error bars represent standard deviations between sample replicates.

To assess whether our Nanotrap particles could, in fact, capture SARS-CoV-2, we infected Vero E6 cells with the virus and incubated for 72 h, allowing the virus to replicate and be released into the cell supernatant, upon which viral titers were measured by plaque assay. We then spiked infectious SARS-CoV-2 at 10 PFU/mL or 1000 PFU/mL into 1 mL of VTM, added Nanotrap particles, and after 30 min of constant agitation, Nanotrap particles were pelleted with a magnet. Pelleted Nanotrap particles were treated with lysis buffer followed by RNA purification on a column. Samples that did not undergo Nanotrap particle processing were used as a control. The results in Fig. 2a and Supplementary Table S1 demonstrate that our method can capture and concentrate infectious SARS-CoV-2 from VTM across multiple virus concentrations and can improve detection of that virus using a real-time RT-PCR assay (IDT).

In recent months, as commercial nucleic acid extraction kits have become supply-chain limited, there have been pre-publication articles showing that it is possible to detect SARS-CoV-2 RNA from samples without an RNA extraction step^15^. As such, we wanted to explore whether our method could be used to improve the detection of SARS-CoV-2 when used with a direct extraction method that utilizes heat and detergent. To accomplish this, we spiked infectious SARS-CoV-2 in 1 mL of VTM, added Nanotrap particles, and after 30 min of constant agitation, Nanotrap particles were pelleted with a magnet. Pelleted Nanotrap particles were either directly treated with lysis buffer followed by RNA purification on a column; or they were mixed with water and heated for 10 min at 95 °C to release the RNA directly into the supernatant; or they were mixed with detergent and heated for 10 min at 95 °C to release the RNA directly into the supernatant. The results in Fig. 2b show that the use of Nanotrap particles improves viral RNA recovery for all tested methods. Interestingly, the yield of the recovered RNA for a heat and detergent method combined with Nanotrap particles is substantially higher than the Nanotrap particle method combined with the column-based RNA purification kits.

Given our success in capturing SARS-CoV-2 from 1 mL sample volumes, we were interested in whether the Nanotrap particles could function just as well in larger volumes of sample; this would improve sample utilization and capture additional viruses from the sample, thereby improving the assay’s ability to detect virus in low viral load samples. To that end, infectious SARS-CoV-2 was spiked into transport medium at 100 PFU/mL. One hundred microliters of this virus were used for RNA extraction and purification with the RNeasy kit. One milliliter of the virus was then mixed with either 4 or 9 mL of additional transport medium. Nanotrap particles were then incubated with the samples for 30 min with constant agitation to capture the virus, and viral extraction was achieved using our direct extraction method. Viral detection was performed using real-time RT-PCR (IDT assay). Results in Fig. 2c indicate that Nanotrap particle processing dramatically improves Ct values in the large volume samples (by 5–6 Ct values) compared to the sample that was not processed with Nanotrap particles. The Ct values in the 5 mL and 10 mL samples were within 1 Ct value of each other, indicating that the Nanotrap particles recovered similar amounts of virus.

### Nanotrap particles capture and concentrate inactivated SARS-CoV-2 from transport medium and saliva

**Figure 3:**
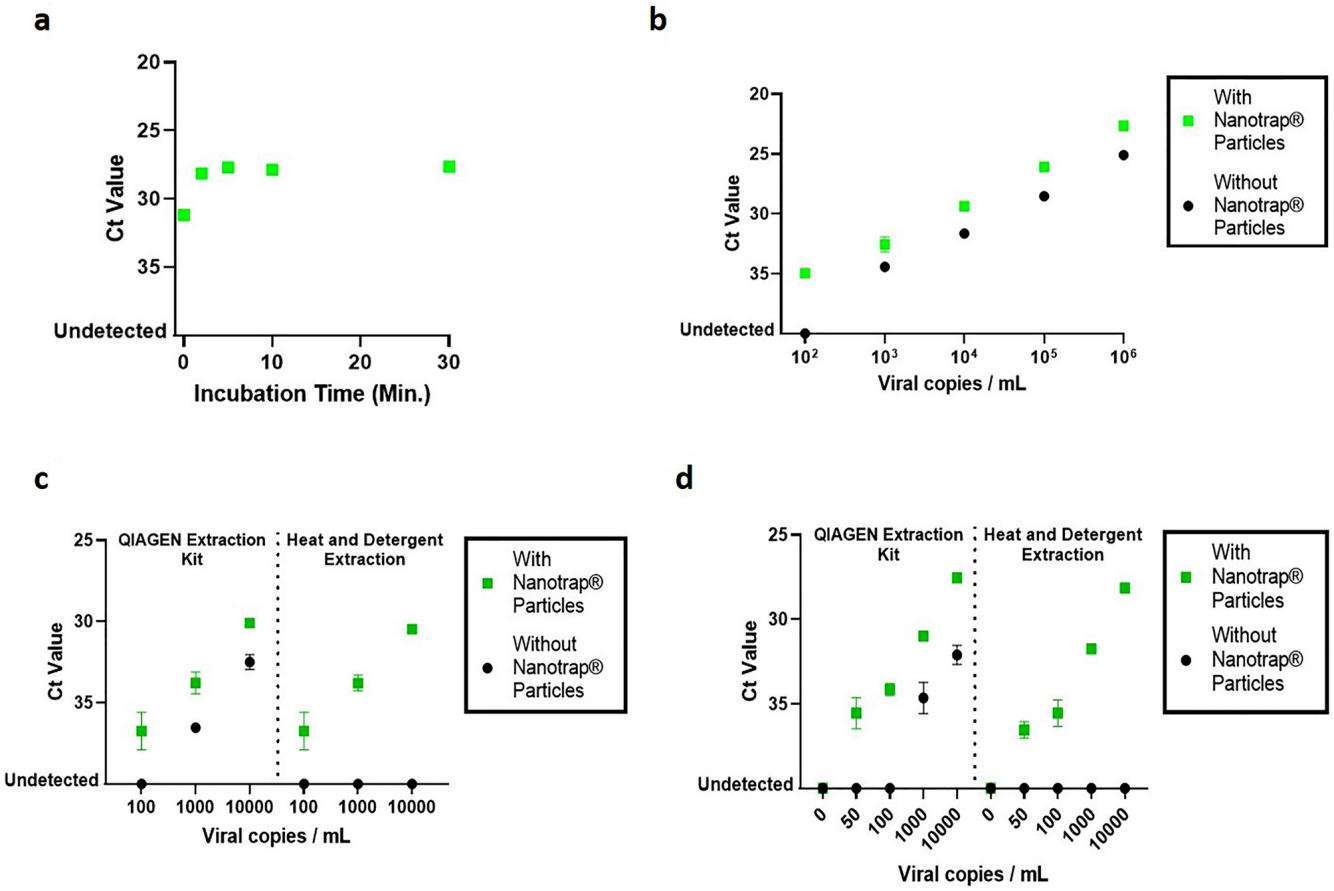
Nanotrap particles rapidly capture heat-inactivated SARS-CoV-2 in multiple sample matrices and improve detection by real-time RT-PCR. (**a**) Heat-inactivated SARS-CoV-2 was spiked into transport medium at 1,000,000 copies/mL. One milliliter samples were added to Nanotrap particles and incubated for various times. Virus was extracted and real-time RT-PCR was performed. A sample without Nanotrap particle processing was used to represent a 0-min incubation with Nanotrap particles. (**b**) Heat inactivated SARS-CoV-2 was spiked into viral transport medium at various titers. One milliliter samples at each viral titer were added to Nanotrap particles and incubated for 5 min. Virus was extracted and real-time RT-PCR was performed. Samples at each titer without Nanotrap particles were used as controls. (**c**) Heat-inactivated SARS-CoV-2 was spiked into transport medium at various titers. One milliliter samples at each viral titer were added to Nanotrap particles and incubated for 5 min. Virus was extracted using multiple methods. Viral detection was performed by real-time RT-PCR. Samples without Nanotrap particles were processed by each extraction method as controls. (**d**) Heat-inactivated SARS-CoV-2 was spiked into saliva at various titers. Saliva aggregates were removed. The liquid saliva was then diluted in PBS/Tween, and 1.8 mL of each dilution was added to Nanotrap particles. After incubating 10 min, the virus was extracted from the Nanotrap particles using multiple extraction methods. Viral detection was performed by real-time RT-PCR. Samples without Nanotrap particles were processed by each extraction method as controls. Error bars represent standard deviations between sample replicates.

Having demonstrated that Nanotrap particles can capture live SARS-CoV-2, we turned our attention to optimizing the workflow with heat-inactive SARS-CoV-2, by reducing the Nanotrap particle incubation time .We spiked 1,000,000 copies/mL of heat-inactivated SARS-CoV-2 (which we found was able to be captured by the Nanotrap particles quite readily; data not shown) into blank transport medium. Then we de-pooled the sample and mixed 1 mL aliquots with Nanotrap particles. The particles were allowed to incubate for either 2, 5, 10, or 30 min with the virus sample. The 2-min and 5-min incubation samples were not agitated during incubation. The 10-min and 30-min samples were inverted once every 5 min to keep the sample mixed, with no additional agitation. A sample without Nanotrap particles was used as a control (the 0-min incubation time point in Fig. 3a). After virus capture, the particles and captured virus were concentrated using a magnet and the supernatant was removed. The pellet was resuspended in PBS and lysis buffer from the QIAamp Viral RNA Mini Kit and incubated for 10 min. Viral nucleic acids were purified and analyzed using the Primerdesign assay as described in the methods section. Results in Fig. 3a and Supplementary Table S2 show that virus capture is fast, as there was no substantial difference in Ct values across the incubation times. Furthermore, capturing and concentrating SARS-CoV-2 from the samples prior to RNA extraction improved Ct values by about 3.

We then asked whether this method could work across a range of viral titers. Heat-inactivated SARS-CoV-2 was spiked into transport medium at 100 copies/mL; 1000 copies/mL; 10,000 copies/mL; 100,000 copies/mL; and 1,000,000 copies/mL. One milliliter aliquots of each viral sample were processed with Nanotrap particles using a 5-min capture time, followed by RNA extraction using the QIAamp Viral RNA Mini Kit. The same extraction kit also was used to extract RNA from the samples without Nanotrap particle processing. Viral RNA was then detected using IDT’s real-time RT-PCR assay. Results in Fig. 3b and Supplementary Table S3 show that capturing and concentrating SARS-CoV-2 prior to viral extraction improves Ct values across all tested viral titers. In samples containing 1000 copies/mL of SARS-CoV-2 or more, the Ct improvement in the Nanotrap particle-processed samples was between 2 and 3. In the sample containing 100 copies/mL, the Nanotrap particles enabled detection of the virus, whereas in the absence of Nanotrap particle concentration of the virus, this sample tested negative in the real-time RT-PCR assay.

We then examined how well our direct extraction method would perform across a range of viral titers. To do so, we spiked transport medium with 100 copies/mL, 1000 copies/mL, or 10,000 copies/mL of heat-inactivated SARS-CoV-2. Samples were processed with and without Nanotrap particles (particle incubation was 5 min), using either the QIAamp Viral RNA Mini Kit or the direct extraction method. Viral detection was performed using IDT’s real-time RT-PCR assay. Results in Fig. 3c and Supplementary Table S4 demonstrate that Nanotrap particles are compatible with both viral extraction methods across the range of viral titers tested and that Nanotrap particles improve Ct values across all viral titers. Interestingly, when used with Nanotrap particles, both extraction methods resulted in similar Ct values for the samples spiked at 100 copies/mL, 1000 copies/mL, and 10,000 copies/mL. We noted that the direct extraction method without Nanotrap particles resulted in no detectable virus across the range of viral titers, which could be due to PCR inhibitors in the transport medium being loaded into the real-time RT-PCR along with the viral nucleic acid^16^.

As swab-based sample collection is technique dependent and because there have been supply chain issues with NP swabs and transport medium, a preprint article indicated that there have been promising efforts to demonstrate that saliva is a useful sample type for SARS-CoV-2 diagnostics^17^. Several groups reportedly have demonstrated that virus is present in saliva samples, and at least one group has already received an EUA from the FDA for a saliva-based COVID-19 test^18^. Thus, we wanted to examine whether our method could be used to capture and concentrate SARS-CoV-2 from saliva samples.

We spiked a pooled saliva sample with varying concentrations of heat-inactivated SARS-CoV-2. We allowed aggregates in the saliva to settle to the bottom of the tube (this process took 2–3 min in a 5 mL saliva sample) prior to taking the supernatant and diluting it in a PBS solution containing a small amount of detergent. We then added Nanotrap particles to 1 mL of the diluted saliva sample and incubated for 10 min at room temperature with an inversion to mix the sample at 5 min. Viral extraction was performed using the QIAamp Viral RNA Mini Kit or the direct extraction method and real-time RT-PCR results (generated by IDT’s assay) were compared to samples that had not undergone Nanotrap particle processing.

The results in Fig. 3d and Supplementary Table S5 demonstrate that capturing and concentrating SARS-CoV-2 from the diluted saliva sample improved real-time RT-PCR results by 3–4 Cts at viral titers of 1000 and 10,000 copies/mL and enabled detection at 50 copies/mL and 100 copies/mL when they were otherwise undetectable. Furthermore, much like the results with transport media samples, Nanotrap particles enabled the use of the direct extraction method.

### Nanotrap particles can eliminate false negatives by concentrating SARS-CoV-2 prior to testing

We next asked whether our method would improve detection of infectious SARS-CoV-2 in samples that had been collected from patients. We obtained two sets of transport medium diagnostic remnant samples that previously had been tested for SARS-CoV-2. The first set of samples came from Discovery Life Sciences and included 9 samples that previously had tested positive and 5 samples that previously had tested negative for SARS-CoV-2 using the Abbott RealTime SARS-CoV-2 EUA test. The second set came from Hancock Regional Hospital in Greenwood, IN. Eight samples previously had tested positive while 27 samples previously had tested negative for SARS-CoV-2 using the Cepheid Xpert Xpress SARS-CoV-2 EUA assay. Each of these samples was processed with and without Nanotrap particles, followed by nucleic acid extraction using the QIAamp Viral RNA Mini Kit and detection by real-time RT-PCR (IDT assay).

**Figure 4:**
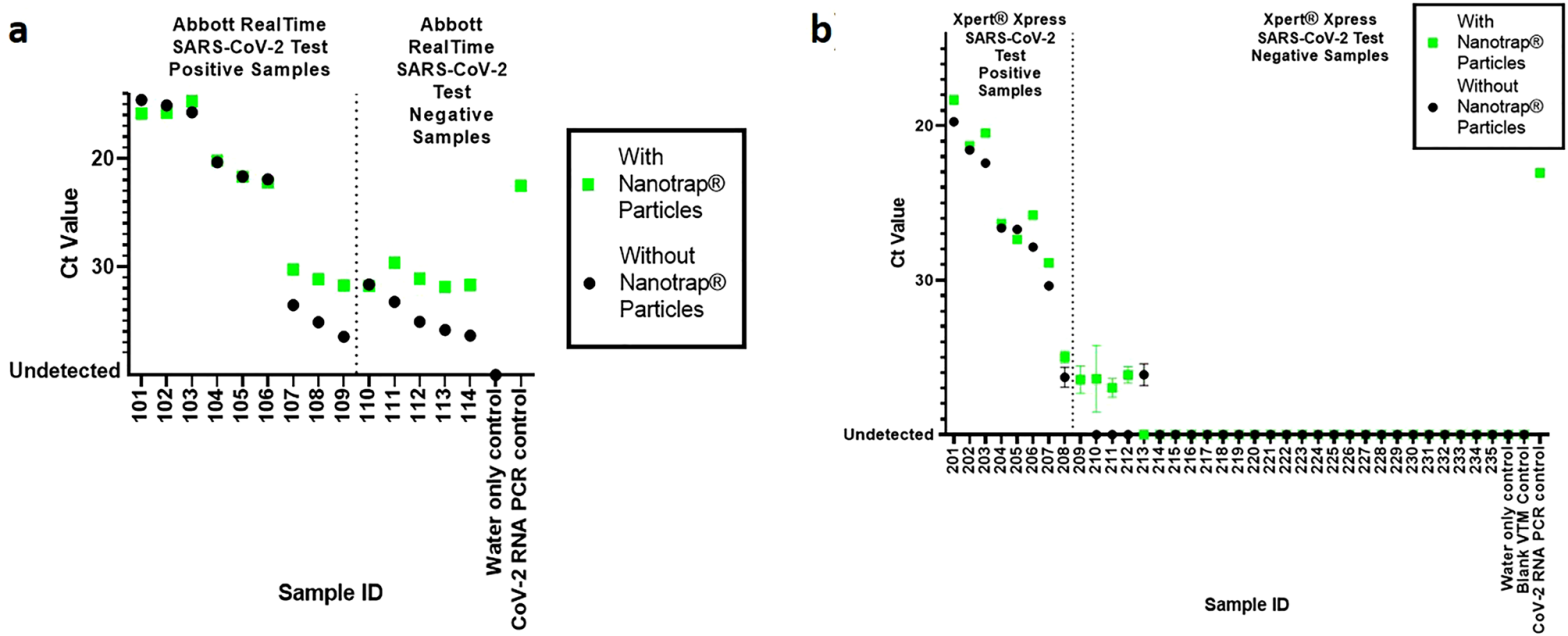
Nanotrap particles improve SARS-CoV-2 detection in low viral load diagnostic remnant samples. Forty-nine swab in transport medium diagnostic remnant samples, (**a**) 14 previously tested for SARS-CoV-2 by the Abbott RealTime SARS-CoV-2 EUA assay (101–114) and (**b**) 35 previously tested for SARS-CoV-2 by the Cepheid Xpert Xpress SARS-CoV-2 EUA assay (201–235), underwent Nanotrap particle processing. One milliliter of each sample was added to Nanotrap particles and incubated for 5 min. Viral extraction was performed, and viral RNA was detected using real-time RT-PCR. Samples 101–109 and Samples 201–208 had previously tested positive for SARS-CoV-2 on the Abbott and Cepheid assays, respectively. Samples 110–114 and Samples 210–235 had previously tested negative. Each diagnostic remnant sample also underwent processing without Nanotrap particles for equivalency with previous results. An uninfected transport medium sample was used as a negative control. Error bars represent standard deviations between sample replicates.

Results in Fig. 4 and Supplementary Tables S6, S7 demonstrate that our method is able to detect SARS-CoV-2 in all 17 samples that had previously tested positive. These results also demonstrate that concentrating virus from transport medium samples prior to RNA extraction substantially improves detection of SARS-CoV-2 in low viral load diagnostic remnant samples as compared to those same samples processed without Nanotrap particles, (average improvement in Ct value when using Nanotrap particles for low viral load samples is 3.1; n = 10). These improvements in Ct value are consistent with the results we saw with our contrived samples.

Of even greater interest to us were the results that suggest that using Nanotrap particles to concentrate the virus from the transport medium prior to nucleic acid extraction can identify potential false negatives. In four of the samples that previously had been reported as negative for SARS-CoV-2 (Samples 209–212 in Fig. 4b), we observed Ct values that were indicative of low levels of virus when we used our method. Note that for Sample 213, Nanotrap particle processing did not result in detection of SARS-CoV-2 even though SARS-CoV-2 was detected in the sample without Nanotrap particle processing (i.e. with QIAamp Viral RNA Mini Kit extraction only). This could be due to sample storage conditions; these samples had been subjected to at least one freeze–thaw cycle, which may have lysed virus in the transport medium prior to the addition of the Nanotrap particles. The Nanotrap particles can capture whole virions but cannot capture free-floating nucleic acids, whereas we assume that a QIAamp Viral RNA Mini Kit extraction kit would capture and purify free-floating viral nucleic acids, regardless of whether those came from previously lysed or recently lysed viruses. No SARS-CoV-2 was observed in our negative control (water), indicating the presence of SARS-CoV-2 in the five previously negative samples is not due to PCR background.

We also noted that the Nanotrap particles did not consistently improve the Ct values of the high viral titer samples (see, for example, Samples 101–106 in Fig. 4a). These results suggest that the Nanotrap particles may become saturated at very high viral titers. This could be addressed by using a larger amount of Nanotrap particles in sample processing, though that may have implications for the very low viral titer samples. For immediate testing ramp-up needs, however, this method appears to be very useful, as getting a very accurate assessment of the viral load is much less clinically relevant than knowing whether virus is present in a sample.

### Nanotrap particles capture and concentrate SARS-CoV-2 from large sample volumes

The diagnostic industry is racing to increase testing capabilities for COVID-19. Despite the impressive efforts thus far, we are still far short of the total number of daily tests that public health experts suggest are necessary^19–21^. To address this shortage, the U.S. Department of Health and Human Services has called for the development of new diagnostic tools, including “pooling of samples from multiple (5, 10, or 20) individuals in a single test” in circumstances where the overall prevalence of the disease is low^22^.

**Figure 5:**
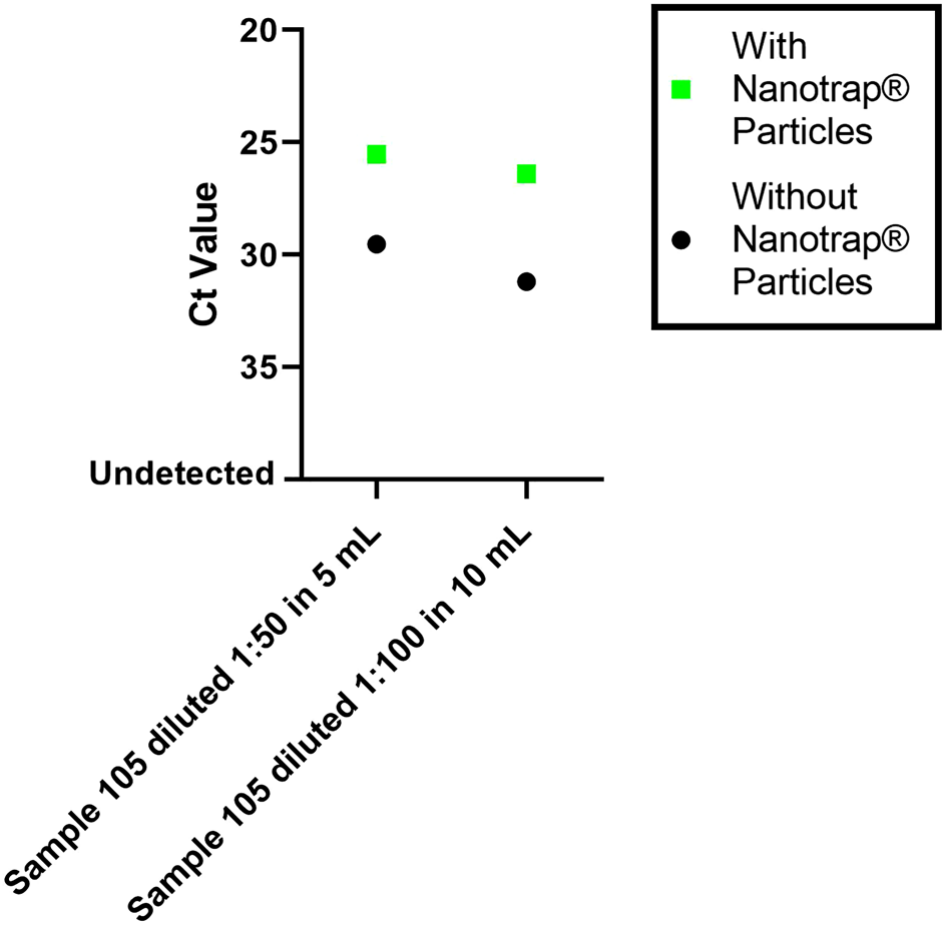
Nanotrap particles improve detection of SARS-CoV-2 from pooled sample mimics. One hundred microliters of SARS-CoV-2-infected viral transport medium from Sample 105 was spiked into uninfected viral transport medium to a total volume of 5 mL or 10 mL. Nanotrap particles were added to the samples and incubated for 5 min. Viral extraction was performed using the QIAamp Viral RNA Mini Kit and SARS-CoV-2 detection was performed by real-time RT-PCR. Samples without Nanotrap particles were processed as controls. Error bars represent standard deviations between sample replicates.

Others have demonstrated that pooling of samples for testing for SARS-CoV-2 can increase test capacity but that it would introduce a risk of borderline positive samples escaping detection^23^. Having already determined that our Nanotrap particles could capture SARS-CoV-2 from large volumes (Fig. 2c), we wanted to investigate whether our method would work for a larger sample volume with a more realistic diagnostic sample. To that end, we diluted 100 µL of diagnostic remnant Sample 105 into uninfected transport medium at 1:50 and 1:100 dilutions (5 mL and 10 mL total volume); each diluted sample contained the same total amount of virus. We added Nanotrap particles to each sample and allowed them to incubate for five minutes with no agitation. Viral extraction was performed using the QIAamp Viral RNA Mini Kit. The 5 mL and 10 mL samples were also processed without Nanotrap particles to serve as controls. The results in Fig. 5 and Supplementary Table S8 show that using Nanotrap particles to capture and concentrate SARS-CoV-2 from 5 and 10 mL transport medium samples substantially improves detection of the virus. Interestingly, the Nanotrap particles were able to recover roughly the same amount of virus in both the 5 mL and 10 mL dilutions, suggesting that the full amount of virus was recovered in both samples, and that even larger sample volumes could be processed using the same method. Collectively, these data indicate that our method could enable much better detection of SARS-CoV-2 in samples that have been pooled prior to RNA extraction. This suggests a path toward testing large numbers of patient pooled samples for presence of SARS-CoV-2 while reducing the reagents and labor needed to perform the nucleic acid extraction and detection steps.

## Discussion

The simple method we have developed can take as little as 10 min to prepare SARS-CoV-2 RNA for downstream testing and obviates the need for commercial nucleic acid extraction kits. Using contrived samples with heat-inactivated and infectious SARS-CoV-2, we have demonstrated substantial improvements in Ct values from transport medium and saliva samples with both commercial nucleic acid extraction kits and a direct extraction method. It is of note that the direct extraction method, which utilizes a detergent solution in water and heat to lyse virus particles and elute viral RNA directly off the Nanotrap particles, allowed for similar or better viral detection by real-time RT-PCR than column-based RNA isolation. This could be because sample loss has been associated with column-based purifications, leading to lower RNA recovery rates^24^. Furthermore, direct extraction is much faster than column-based extraction, taking 75% less time (5 min extraction for direct extraction vs. 10 min extraction and 15 min purification for column-based extraction). This allows Nanotrap particles to enable direct extraction as a viable alternative to column-based extraction.

Contrived samples lack the biological complexity (i.e. cells, cell debris, bacteria) contained in patient samples. Using 49 diagnostic remnant samples, we demonstrated that our method can improve detection of SARS-CoV-2 in real samples and eliminate false negatives. Our method had 100% concordance with all of the samples that previously tested positive, improved the real-time RT-PCR signal by an average of 3.1 Ct values in the low viral load samples, and identified SARS-CoV-2 in four of the samples that had previously tested negative.

The national and international targets for numbers of daily tests are astronomical. There is a growing consensus that pooling of samples must be implemented in order to reach those numbers of daily tests, an opinion which is shared by the government of the United States^23,25^. Our method was able to concentrate infectious SARS-CoV-2 from contrived samples and diagnostic remnant samples in pooled patient sample mimics, an approach which is a promising way forward to address the massive testing scale-ups that are necessary.

There are several limitations to this study. First, we only had 14 diagnostic remnant samples that had been previously tested on the Abbott RealTime SARS-CoV-2 EUA assay, and only five of those samples had previously tested negative for SARS-CoV-2. A larger set of samples would help us assess how our method compares to that assay. Second, for all of the diagnostic remnant samples that we used, the storage conditions were inconsistent. Some samples were unintentionally subjected to multiple freeze–thaw cycles, others were only subjected to one free-thaw cycle, and others were shipped and stored at 4 ℃ and never frozen. Third, it is important to note that we used a different detection assay (from IDT) than had been used previously on the diagnostic remnant samples; therefore, we cannot make any direct conclusions regarding whether Nanotrap particles would improve the performance of those assays. We did not test the Nanotrap particles in bronchoalveolar lavages, which are a common sample matrix for diagnosis of COVID-19, though we are aware of at least one instance of Nanotrap particles being used successfully to extract RNA from such samples (https://www.fda.gov/media/136151/download). Additionally, our saliva data is limited to contrived samples and it is therefore difficult to gauge how effective our method would be for saliva SARS-CoV-2 testing in a clinical setting.

Despite these limitations, we are confident that the method described here can offer increased sensitivity for SARS-CoV-2 molecular testing from transport medium and saliva while enabling the use of faster and easier nucleic acid extraction methods, which will help address supply chain limitations. Also, as we have previously demonstrated that the Nanotrap particles used in this study can also capture and concentrate influenza viruses, RSV, and other coronaviruses^14^, we are confident that the method described here will be compatible with the respiratory virus panel tests that are under development. Moreover, while we did not address assays for viral antigens, next generation sequencing assays, or antibody assays in this work, Nanotrap particles can be used to improve those testing modalities^11,12,26–28^ and we look forward to expanding our work into that area.

## Methods and materials

### Cells, viruses, and reagents

Vero E6 cells were obtained from ATCC (CRL-1586) and were grown in DMEM complete medium, consisting of 10% FBS, 5% l-glutamine, and 5% penicillin/streptomycin, at 37 °C and 5% CO_2_. Infectious, replication-competent and heat-inactivated SARS-CoV-2 were obtained from BEI Resources (NR-52281 and NR-52286, respectively). For transport medium experiments, Puritan UniTranz-RT Universal Transport Medium (UT-300) was utilized. Nanotrap Magnetic Virus Particles (SKU 44202) were manufactured by the authors at Ceres Nanosciences Inc and utilized by authors at both Ceres and George Mason University. Dulbecco’s phosphate-buffered Saline Solution without Ca^2+^ and Mg^2+^ (PBS) was used during viral extraction. TRIzol LS was purchased from ThermoFisher Scientific (Cat. #10296010). Patient pooled saliva was purchased from BIOIVT (Saliva-1902492).

### Diagnostic remnant samples

Fourteen diagnostic remnant samples of patient swabs in viral transport media were purchased from Discovery Life Sciences. Each sample previously had been tested for SARS-CoV-2 using the Abbott RealTime SARS-CoV-2 test. Nine samples (sample ID’s 101–109) previously had tested positive. Five samples (sample ID’s 110–114) had previously tested negative. An additional 35 diagnostic remnant samples were provided by Hancock Regional Hospital in Greenwood, IN, all of which had been previously tested for SARS-CoV-2 using the Cepheid Xpert Xpress SARS-CoV-2 test. Eight samples (sample ID’s 201–208) had previously tested positive for COVID-19. Twenty-seven had previously tested negative (sample ID’s 209–235). Most of the samples were frozen after testing at the original sites and were shipped frozen. Due to challenges associated with shipping, a few of the samples were never frozen and were shipped and received refrigerated and at least one sample underwent multiple freeze–thaw cycles. All frozen samples were thawed directly prior to processing. All samples were from human biological material leftover from previous diagnostic testing and were de-identified to protect patient confidentiality. Under OHRP definition 45 CFR 46.102(d)(f), these coded specimens are not considered human research subjects and require no informed consent.

### Concentrating SARS-CoV-2 from transport medium

SARS-CoV-2 (heat-inactivated or infectious, replication-competent) was spiked into Puritan Universal Transport Medium at various concentrations. Two hundred microliters (1 mg) of Nanotrap particles in the storage solution were added to 1.5 mL microcentrifuge tubes and pulled out of solution using a DynaMag-2 magnet from ThermoFisher (12321D). The supernatant was removed, and 1 mL of spiked transport medium was added to the particles; a quick mixing of the sample and the Nanotrap particles was performed using a micropipette. For large volume samples (5 or 10 mL of spiked transport medium), 300 mL (1.5 mg) of Nanotrap particles were used. Unless otherwise described, samples were incubated with Nanotrap particles at room temperature for 5 min to capture virus. Other than the initial mixing of the sample and the particles, no mixing was required during the 5-min incubation. For incubations longer than 5 min, samples were inverted once every 5 min. Following incubation, Nanotrap particles were pulled out of solution with the DynaMag-2 magnet and the supernatant was discarded.

### Concentrating SARS-CoV-2 from saliva

Heat-inactivated SARS-CoV-2 was spiked into the saliva at various concentrations. The saliva was allowed to sit for 3 min while aggregates settled. The liquid phase of saliva, which contained the virus, was moved into a new tube and was diluted in PBS with 0.05% Tween-20 (one-part saliva was added to two-parts PBS/Tween). Aliquots of 1.8 mL of diluted saliva were added to three hundred microliters (1.5 mg) of Nanotrap particles, which previously had been removed from their storage solution using a DynaMag-2 magnetic rack. Samples were incubated at room temperature for 10 min (at the 5-min mark, samples were inverted once). Following incubation, Nanotrap particles were pulled out of solution with a DynaMag-2 magnet and the supernatant was discarded.

### Nucleic acid extraction

We evaluated the impact of using Nanotrap particles to capture and concentrate SARS-CoV-2 on several nucleic acid extraction methods: the QIAamp Viral RNA Mini Kit from QIAGEN (52906); the RNeasy Mini Kit from QIAGEN (74106); a TRIzol LS method used in combination with the RNeasy Mini Kit; a heat extraction method, which used a short heating step to lyse the virus; and a heat and detergent method, which used a short heating step and some detergent to lyse the virus. Those extraction methods are described below in their uses with and without Nanotrap particles.

When Nanotrap particles were used to capture and concentrate virus prior to nucleic acid extraction, nucleic acids were extracted under the following conditions. For the QIAamp Viral RNA Mini Kit, the Nanotrap particle pellet was resuspended in 150 µL of PBS and 560 µL of viral lysis buffer (Buffer AVL) was added to the re-suspended particles and allowed to incubate for 10 min at room temperature. Following this, particles were pulled out of solution using the DynaMag-2 magnet, and the supernatant was collected and added to 560 µL of ethanol, upon which the manufacturer’s protocol was followed without any deviations.

For the TRIzol LS method, the Nanotrap particle pellet was resuspended in 100 µL of water, mixed with 300 µL of TRIzol LS for virus inactivation, and incubated for 10 min at room temperature. This was followed by mixing with 200 µL of chloroform, spinning down, collecting the upper phase, mixing the upper phase with 300 µL of RLT buffer from the RNeasy Mini Kit, and adding an equal volume of 70% ethanol to the mixture. The next purification steps were followed according to the manufacturer’s protocol for the RNeasy Mini Kit.

For the direct extraction method, the Nanotrap particle pellet was resuspended in a detergent buffer and was incubated for 5 min at 95 °C. For heat only extraction, the Nanotrap particle pellet was resuspended in 50 µL of PCR grade water and heated for 10 min at 95 °C. Following this, the particles were pulled out of solution using the DynaMag-2 magnet, and the supernatant was collected for analysis. At the end of each of these methods, the resulting solution, with extracted RNA, was loaded into the downstream assay.

In samples processed without Nanotrap particles, nucleic acids were extracted under the following conditions. For the QIAamp Viral RNA Mini Kit, nucleic acids were extracted from 150 µL of sample according to the manufacturers’ protocol.

For the RNeasy Mini Kit, nucleic acids were extracted from 150 µL of sample according to the manufacturers’ protocol.

For the TRIzol LS method, 100 µL of sample was mixed with 300 µL of TRIzol LS for virus inactivation. After that samples were mixed with 200 µL of chloroform, spun down, the upper phase was mixed with 300 µL RLT buffer from the RNeasy Mini Kit, and an equal volume of 70% ethanol was added. This was followed by loading samples onto the column and RNA was purified according to the RNeasy Mini Kit recommendations.

For the direct extraction method, 50 µL of sample was heated at 95 °C for ten minutes. At the end of each of these extraction methods, the resulting solution, with extracted RNA, was loaded into the downstream assay.

### Real-time RT-PCR

For heat-inactivated SARS-CoV-2 samples, two assays, the Primerdesign Ltd COVID-19 Genesig Real-Time CE-IVD/EUA PCR assay (Z-COVID-19) and the IDT 2019 nCoV CDC EUA Kit (1006770), were used for real-time RT-PCR. Per Primerdesign’s instructions, 10 µL of the Oasig™ OneStep 2X qRT-PCR Master Mix, 2 µL of COVID-19 primer/probe, and 8 µL of RNA template were added to each well. PCR conditions were performed according to Primerdesign’s instructions on a Roche LightCycler 96. All samples were run in technical triplicate. Resulting Ct values were averaged across replicates and standard deviations were calculated.

Per IDT’s recommendation, TaqPath 1-Step RT-qPCR Master Mix from ThermoFisher (A15300) was used in the IDT 2019 nCoV CDC EUA assay. Per IDT’s instructions, each PCR reaction used 8.5 µL of nuclease free water, 5 µL of the TaqPath solution, 1.5 µL of the N1 primer/probe, and 5 µL of RNA template. PCR conditions were performed according to IDT’s instructions on a Roche LightCycler 96. All samples were run in technical triplicate. Resulting Ct values were averaged across replicates and standard deviations were calculated. All heat-inactivated SARS-CoV-2 experiments utilized this assay unless otherwise denoted. All patient remnant samples utilized this assay.

For infectious, replication-competent SARS-CoV-2 samples, we used the N1 primer from the IDT 2019 nCoV CDC EUA Kit (1006770). For the real-time RT-PCR assay, we used the RNA UltraSense™ One-Step Quantitative RT-PCR System from Applied Biosystem (11732927) according to manufacturer’s recommendation. Each PCR reaction contained 1 µL UltraSense Enzyme Mix, 5 µL RNA UltraSense 5X Reaction Mix, 1.5 µL of primer/probe mixture from IDT, 0.4 µL ROX Reference Dye, 8.1 µL of nuclease free water, and 5 µL of RNA template. The real-time RT-PCR was performed using an ABI StepOne Plus instrument. All samples were run in biological triplicate unless otherwise described.

## Supplementary Information

Supplementary Information

## References

[1] Decaro N, Lorusso A (2020). Novel human coronavirus (SARS-CoV-2): A lesson from animal coronaviruses. Vet. Microbiol..

[2] Guan WJ (2020). Clinical characteristics of coronavirus disease 2019 in China. N. Engl. J. Med..

[3] Chen N (2020). Epidemiological and clinical characteristics of 99 cases of 2019 novel coronavirus pneumonia in Wuhan, China: A descriptive study. Lancet.

[4] Mahase E (2020). China coronavirus: WHO declares international emergency as death toll exceeds 200. BMJ.

[5] Dong E, Du H, Gardner L (2020). An interactive web-based dashboard to track COVID-19 in real time. Lancet Infect. Dis..

[6] Corman VM (2020). Detection of 2019 novel coronavirus (2019-nCoV by real-time RT-PCR. Euro Surveill..

[7] Mullegama S (2019). Nucleic acid extraction from human biological samples. Methods Mol. Biol..

[8] C&EN. https://cen.acs.org/analytical-chemistry/diagnostics/Shortage-RNA-extraction-kits-hampers/98/web/2020/03. (2020).

[9] Pfefferle S, Reucher S, Norz D, Lutgehetmann M (2020). Evaluation of a quantitative RT-PCR assay for the detection of the emerging coronavirus SARS-CoV-2 using a high throughput system. Euro Surveill..

[10] Wang X (2020). Limits of detection of six approved RT-PCR kits for the novel SARS-coronavirus-2 (SARS-CoV-2). Clin. Chem..

[11] Akhrymuk I (2019). Magnetic nanotrap particles preserve the stability of venezuelan equine encephalitis virus in blood for laboratory detection. Front. Vet. Sci..

[12] Shafagati N (2014). The use of nanotrap particles for biodefense and emerging infectious disease diagnostics. Pathog. Dis..

[13] Shafagati N (2013). The use of NanoTrap particles as a sample enrichment method to enhance the detection of Rift Valley Fever Virus. PLoS Negl. Trop. Dis..

[14] Shafagati N (2016). Enhanced detection of respiratory pathogens with nanotrap particles. Virulence.

[15] Bruce, E. A. *et al.* Direct RT-qPCR detection of SARS-CoV-2 RNA from patient nasopharyngeal swabs without an RNA extraction STEP. Preprint at https://www.biorxiv.org/content/10.1101/2020.03.20.001008v2 (2020) 10.1371/journal.pbio.3000896 PMC7556528 33006983

[16] Buckwalter SP (2014). Inhibition controls for qualitative real-time PCR assays: Are they necessary for all specimen matrices?. J. Clin. Microbiol..

[17] Wyllie, A. L., et al. Saliva is more sensitive for SARS-CoV-2 detection in COVID-19 patients than nasopharyngeal swabs. Preprint at: https://www.medrxiv.org/content/10.1101/2020.04.16.20067835v1 (2020).

[18] FDA. Rutgers Clinical Genomics Laboratory TaqPath SARS-CoV-2 Assay. https://www.fda.gov/media/137773/download (2020).

[19] Jha A. K., J. B., Friedhoff, S., & Tsai, T. HGHI and NPR publish new state testing targets. https://globalepidemics.org/2020/05/07/hghi-projected-tests-needed-may15/ (2020).

[20] The COVID Tracking Project. https://covidtracking.com/ (2020).

[21] Allen, D., et al. *Roadmap to Pandemic Resilience*. https://ethics.harvard.edu/files/center-for-ethics/files/roadmaptopandemicresilience_updated_4.20.20_1.pdf (2020).

[22] DHHS. Report to Congress: COVID-19 Strategic Testing Plan. https://www.democrats.senate.gov/imo/media/doc/COVID%20National%20Diagnostics%20Strategy%2005%2024%202020%20v%20FINAL.pdf (2020).

[23] Lohse S (2020). Pooling of samples for testing for SARS-CoV-2 in asymptomatic people. Lancet Infect. Dis..

[24] Lloyd L, Macgregor B, Teske A (2010). Quantitative PCR methods for RNA and DNA in marine sediments: Maximizing yield while overcoming inhibition. FEMS Microbiol. Ecol..

[25] Hogan CA, Sahoo MK, Pinsky BA (2020). Sample pooling as a strategy to detect community transmission of SARS-CoV-2. J. Am. Med. Assoc..

[26] Shafagati N (2015). The use of Nanotrap particles in the enhanced detection of Rift Valley fever virus nucleoprotein. PLoS One.

[27] Keller, M. Portable influenza A virus diagnostics and surveillance. *AGBT 2020* (2020).

[28] Levandoski, M., & Cartwright, C. Purification and conservation of CMV antibody in oral fluid using nanotrap particle technology. *2017 ASM Clinical Virology Symposium* (2017).

