# Supplementary material for: Hydrogel particles improve detection of SARS-CoV-2 RNA from multiple sample types
Source: Sci Rep. 2020 Dec 30;10:22425. doi: 10.1038/s41598-020-78771-8 (PMC7773739; doi:10.1038/s41598-020-78771-8)
Supplement: Supplementary file 1 — Supplementary Information [file 41598_2020_78771_MOESM1_ESM.pdf]

Hydrogel particles improve detection of SARS-CoV-2 for pooled sample methods, extraction-free saliva methods, and extraction-free transport medium methods

Barclay RA[1]; Akhrymuk I[2]; Patnaik A[1]; Callahan V[2]; Lehman C [2]; Andersen P[1]; Barbero R[1]; Barksdale S[1]; Dunlap R[1]; Goldfarb D[1]; Jones-Roe T[1]; Kelly R[1]; Kim B[1]; Miao S[1]; Munns A[1]; Munns D[1]; Patel S[1]; Porter E[1]; Ramsey R[1]; Sahoo S[1]; Swahn O[1]; Warsh J[1]; Kehn-Hall K[2]; Lepene B[1*]

Corresponding Author Email: blepene@ceresnano.com

1. Ceres Nanosciences, Inc., Manassas, VA 20110

2. National Center for Biodefense and Infectious Diseases, School of Systems Biology, George Mason University, Manassas, VA 20110

a)

Copies/mL	NT- Ct	NT+ Ct
100	Undetected	36.75
1,000	36.54	33.78
10,000	32.5	30.09

b)

Copies/mL	NT- Ct	NT+ Ct
100	Undetected	36.75
1,000	Undetected	33.79
10,000	Undetected	30.47

c)

Sample	Ct
Infectious Virus	29.82
1:5	24.84
1:10	23.93

Supplementary Table 1: Nanotrap® particles capture infectious SARS-CoV-2 in contrived samples. This table shows Ct values plotted in **Fig. 2a** (**a**), **Fig. 2b** (**b**) and **Fig. 2c** (**c**).

Time (Min)	Ct Value (Average)	Ct Value (Standard Deviation)
0	31.17	0.14
2	28.15	0.08
5	27.70	0.00
10	27.87	0.04
30	27.65	0.08

Supplementary Table 2: Nanotrap® particles capture SARS-CoV-2 in as little as two minutes. This table shows the Ct values plotted in **Fig. 3a**.

Copies/mL	Ct Value without Nanotrap particles (Average)	Ct Value without Nanotrap particles (Standard Deviation)	Ct Value with Nanotrap particles (Average)	Ct Value with Nanotrap particles (Standard Deviation)
0	Undetected	N/A	Undetected	N/A
100	Undetected	N/A	34.96	0.36
1,000	34.42	0.32	32.56	0.63
10,000	31.64	0.09	29.37	0.11
100,000	28.53	0.02	26.08	0.04
1,000,000	25.09	0.04	22.65	0.01

Supplementary Table 3: Nanotrap® particles capture SARS-CoV-2 across a range of titers. This table shows the Ct values plotted in **Fig. 3b**.

a)

Copies/mL	Ct Value without Nanotrap particles (Average)	Ct Value without Nanotrap particles (Standard Deviation)	Ct Value with Nanotrap particles (Average)	Ct Value with Nanotrap particles (Standard Deviation)
100	Undetected	N/A	36.75	1.16
1,000	36.54	0.08	33.78	0.67
10,000	32.50	0.46	30.09	0.22

b)

Copies/mL	Ct Value without Nanotrap particles (Average)	Ct Value without Nanotrap particles (Standard Deviation)	Ct Value with Nanotrap particles (Average)	Ct Value with Nanotrap particles (Standard Deviation)
100	Undetected	N/A	36.75	1.16
1,000	Undetected	N/A	33.79	0.50
10,000	Undetected	N/A	30.47	0.12

Supplementary Table 4: Nanotrap® particles compatible with multiple viral extraction methods in transport medium. This table shows the Ct values plotted in **Fig. 4c** for commercial kit extraction (**a**) and direct extraction (**b**).

a)

Copies/mL	Ct Value without Nanotrap particles (Average)	Ct Value without Nanotrap particles (Standard Deviation)	Ct Value with Nanotrap particles (Average)	Ct Value with Nanotrap particles (Standard Deviation)
0	Undetected	N/A	Undetected	N/A
50	Undetected	N/A	35.55	0.92
100	Undetected	N/A	34.15	0.35
1,000	34.65	0.92	31.00	0.00
10,000	32.10	0.57	27.55	0.07

b)

Copies/mL	Ct Value without Nanotrap particles (Average)	Ct Value without Nanotrap particles (Standard Deviation)	Ct Value with Nanotrap particles (Average)	Ct Value with Nanotrap particles (Standard Deviation)
0	Undetected	N/A	Undetected	N/A
50	Undetected	N/A	36.55	0.49
100	Undetected	N/A	35.55	0.78
1,000	Undetected	N/A	31.75	0.07
10,000	Undetected	N/A	28.15	0.07

Supplementary Table 5: Nanotrap® particles compatible with multiple viral extraction methods in saliva. This table shows the Ct values plotted in **Fig. 3d** for commercial kit extraction (**a**) and direct extraction (**b**).

Sample	Ct Value without Nanotrap particles (Average)	Ct Value without Nanotrap particles (Standard Deviation)	Ct Value with Nanotrap particles (Average)	Ct Value with Nanotrap particles (Standard Deviation)
101	14.55	0.21	15.81	0.05
102	15.05	0.09	15.75	0.06
103	15.69	0.28	14.68	0.15
104	20.33	0.04	20.18	0.09
105	21.65	0.13	21.67	0.04
106	21.90	0.08	22.19	0.05
107	33.53	0.31	30.24	0.11
108	35.11	0.01	31.15	0.10
109	36.46	0.37	31.71	0.03
110	31.62	0.12	31.75	0.12
111	33.23	0.18	29.60	0.17
112	35.08	0.36	31.08	0.06
113	35.82	0.06	31.85	0.04
114	36.36	0.37	31.68	0.04

Supplementary Figure 6: Nanotrap® particles capture live SARS-CoV-2 in diagnostic remnant samples previously tested by the Abbot RealTime SARS-CoV-2 EUA Test. This table shows Ct values plotted in **Fig. 4a**.

Sample	Ct Value without Nanotrap particles (Average)	Ct Value without Nanotrap particles (Standard Deviation)	Ct Value with Nanotrap particles (Average)	Ct Value with Nanotrap particles (Standard Deviation)	Sample	Ct Value without Nanotrap particles (Average)	Ct Value without Nanotrap particles (Standard Deviation)	Ct Value with Nanotrap particles (Average)	Ct Value with Nanotrap particles (Standard Deviation)
201	19.72	0.06	18.32	0.11	218	Undetected	N/A	Undetected	N/A
202	21.55	0.06	21.29	0.10	219	Undetected	N/A	Undetected	N/A
203	22.41	0.04	20.45	0.15	220	Undetected	N/A	Undetected	N/A
204	26.61	0.08	26.32	0.08	221	Undetected	N/A	Undetected	N/A
205	26.70	0.06	27.34	0.13	222	Undetected	N/A	Undetected	N/A
206	27.85	0.05	25.78	0.12	223	Undetected	N/A	Undetected	N/A
207	30.36	0.17	28.87	0.07	224	Undetected	N/A	Undetected	N/A
208	36.28	0.63	34.97	0.37	225	Undetected	N/A	Undetected	N/A
209	Undetected	N/A	36.43	0.86	226	Undetected	N/A	Undetected	N/A
210	Undetected	N/A	36.39	2.15	227	Undetected	N/A	Undetected	N/A
211	Undetected	N/A	36.96	0.60	228	Undetected	N/A	Undetected	N/A
212	Undetected	N/A	36.13	0.51	229	Undetected	N/A	Undetected	N/A
213	36.12	0.70	Undetected	N/A	230	Undetected	N/A	Undetected	N/A
214	Undetected	N/A	Undetected	N/A	231	Undetected	N/A	Undetected	N/A
215	Undetected	N/A	Undetected	N/A	232	Undetected	N/A	Undetected	N/A
216	Undetected	N/A	Undetected	N/A	233	Undetected	N/A	Undetected	N/A
217	Undetected	N/A	Undetected	N/A	234	Undetected	N/A	Undetected	N/A

Supplementary Figure 7: Nanotrap® particles capture live SARS-CoV-2 in diagnostic remnant samples previously tested by the Cepheid Xpert® Xpress SARS-CoV-2 EUA assay. This table shows Ct values plotted in **Fig. 4b**.

Sample	NT- Ct	NT+ Ct
1:50	29.37	25.52
1:100	31.25	26.39

Supplementary Table 8: Nanotrap® particles improve SARS-CoV-2 detection in large volume samples. This table shows Ct values plotted in **Fig. 5a** (**a**) and **Fig. 5b** (**b**).
